# Efficacy of Melatonin in Metabolic Dysfunction-Associated Steatotic Liver Disease: A Systematic Review and Meta-Analysis

**DOI:** 10.14740/jocmr6688

**Published:** 2026-10-23

**Authors:** Avneet Singh, Fariha Hasan, Marium Zahid, Syeda Hafsa Qadri, Mohammed Aloqaily, Zehara Abidi, Alexa Conner, Alexander Garcia, Dushyant Singh Dahiya, Krysta Contino, Christina Tofani, Anthony Infantolino, Satyajeet Roy

**Affiliations:** a Department of Medicine, Cooper University Health Care, Camden, NJ, USA; b Karachi Medical and Dental College, North Nazimabad, Karachi, Sindh, Pakistan; c Department of Medicine, Dow University of Health Sciences, Karachi, Sindh, Pakistan; d Department of Medicine, University of Maryland Midtown, Baltimore, MD, USA; e Department of Medicine, Touro University, New York, NY, USA; f New York Institute of Technology College of Osteopathic Medicine, Old Westbury, NY, USA; g The University of Kansas School of Medicine, Kansas City, KS, USA; h Cooper Medical School of Rowan University, Camden, NJ, USA

**Keywords:** Metabolic dysfunction-associated steatotic liver disease, Fatty liver, Hepatic fibrosis, Melatonin

## Abstract

**Background:**

Metabolic dysfunction-associated steatotic liver disease (MASLD) represents the leading cause of chronic liver disease globally. Oxidative stress, inflammation, and hepatocellular necrosis play central roles in its pathogenesis. Emerging evidence highlights melatonin as a promising therapeutic agent for MASLD. This systematic review and meta-analysis aimed to evaluate the efficacy of melatonin in MASLD management.

**Methods:**

A comprehensive literature search was conducted across PubMed, Embase, Cochrane Library, and Web of Science from inception through December 2025 for randomized and non-randomized controlled clinical trials comparing melatonin supplementation with placebo, no treatment, or an active comparator in adults with MASLD. Dichotomous outcomes were pooled as risk ratios (RRs) using the Mantel–Haenszel method and continuous outcomes as mean differences (MDs) using the inverse-variance method, both under random-effects models. In every trial the control arm received the same background management as the melatonin arm (placebo, essential phospholipids, statin therapy, or lifestyle advice) without melatonin; no trial compared melatonin with an active pharmacological comparator.

**Results:**

Eleven controlled trials (nine randomized, two non-randomized; 548 participants: 300 melatonin, 248 control) were included. In the primary analyses, melatonin was associated with reductions in total cholesterol (MD: −16.80 mg/dL, 95% confidence interval (CI): −29.13 to −4.47, P = 0.008; I^2^ = 76%) and low-density lipoprotein (LDL) cholesterol (MD: −52.4 mg/dL, 95% CI: −100.52 to −4.27, P = 0.03; I^2^ = 96%), in interleukin-1 (IL-1), interleukin-6 (IL-6), and tumor necrosis factor-alpha (TNF-α), and in body weight and waist circumference, and with a lower proportion of participants with grade III steatosis at follow-up (RR: 0.23, 95% CI: 0.07–0.77; two trials). No significant differences were found for grade I or II steatosis, alanine aminotransferase (ALT), aspartate aminotransferase (AST), gamma-glutamyl transferase (GGT), alkaline phosphatase (ALP), triglycerides, high-density lipoprotein (HDL), fasting blood sugar, systolic/diastolic blood pressure, or high-sensitivity C-reactive protein (hs-CRP). Reductions in ALT, AST, GGT, and triglycerides were observed only in exploratory sensitivity analyses after exclusion of individual trials. Heterogeneity was high (I^2^ > 75%) for several outcomes, and nine of the 11 trials were placebo-controlled.

**Conclusion:**

Melatonin supplementation was associated with modest improvements in total and LDL cholesterol, selected inflammatory markers, and anthropometric measures, but not in liver enzymes or grade I–II steatosis. Given the small number of trials, the inclusion of non-randomized designs, high heterogeneity, heterogeneous comparators, and the exploratory nature of the sensitivity analyses, these findings are preliminary. Melatonin may merit evaluation as an adjunctive therapy in MASLD, but larger, well-designed randomized trials with standardized comparators and systematic safety reporting are required.

## Introduction

Metabolic dysfunction-associated steatotic liver disease (MASLD) is defined by hepatic steatosis in the presence of at least one cardiometabolic risk factor: overweight or obesity, type 2 diabetes, hypertension, hypertriglyceridemia, or low high-density lipoprotein (HDL) cholesterol, and in the absence of significant alcohol intake or other causes of steatosis [1, 2]. Although MASLD shares its metabolic risk factors with, and frequently coexists with, the metabolic syndrome, it is a distinct hepatic disease entity [1, 3]. MASLD is the most prevalent cause of chronic liver disease, affecting approximately 30% of the population in the United States and globally, with incidence rates continuing to rise [1–4]. Notably, obesity is strongly associated with MASLD, and approximately 66% of adults with obesity develop hepatic steatosis [1]. Typically, patients with hepatic steatosis exhibit no symptoms, and the condition is often identified through laboratory tests or incidentally during imaging studies [1].

Multiple mechanisms are implicated in the development of MASLD, including oxidative stress, inflammatory responses, insulin resistance, hepatic cell necrosis and apoptosis, and fat tissue infiltration with subsequent inflammation [1, 3, 4]. These underlying processes provide a foundation for developing targeted treatments [2, 3]. The importance of diagnosing and managing MASLD lies in its potential to progress along a spectrum from simple steatosis to metabolic dysfunction-associated steatohepatitis (MASH), advanced fibrosis, cirrhosis, and hepatocellular carcinoma, and in its strong association with cardiovascular disease, which is the leading cause of death in affected patients [1, 4]. Despite being the most prevalent form of chronic liver disease, the management of MASLD primarily involves lifestyle modifications, such as dietary changes and regular physical activity, in addition to controlling comorbid conditions such as hypertension, diabetes, and hypercholesterolemia [1, 4]. Several of these pathogenic processes are plausible targets for melatonin. Melatonin and its metabolites directly scavenge reactive oxygen species and up-regulate antioxidant enzymes such as glutathione peroxidase and superoxide dismutase [5, 6]; it attenuates nuclear factor kappa B (NF-κB)-dependent production of pro-inflammatory cytokines including interleukin-1 (IL-1), interleukin-6 (IL-6), and tumor necrosis factor-alpha (TNF-α) [3, 4]; and, through MT1/MT2 receptor signaling and its circadian actions, it modulates insulin sensitivity, hepatic *de novo* lipogenesis, and adipose-tissue lipid mobilization [7–9]. These properties provide the biological rationale for evaluating melatonin in MASLD.

Melatonin, a neuroendocrine hormone primarily secreted by the pineal gland, is also produced by other organs such as the liver and bone marrow [3, 4, 7]. Melatonin is well-recognized for its roles in regulating the sleep cycle, exerting anti-inflammatory effects, and serving as a protective agent against oxidative stress and free radicals [3, 4, 7]. Furthermore, studies have shown that melatonin influences glucose and lipid metabolism [3, 4, 7].

Although several experimental and clinical studies have evaluated melatonin in MASLD [3, 4, 7], the evidence remains fragmented and heterogeneous. Prior reviews have combined preclinical, observational, and interventional evidence, limiting the ability to isolate the effect of melatonin supplementation. A systematic synthesis restricted to controlled clinical trials in humans, with separate appraisal of randomized and non-randomized designs, is therefore needed to clarify the clinical efficacy of melatonin in MASLD. This systematic review and meta-analysis aimed to evaluate the impact of melatonin supplementation on hepatic, metabolic, inflammatory, and anthropometric outcomes in patients with MASLD. The primary research question was: in adults with MASLD (population), does melatonin supplementation (intervention), compared with placebo, no treatment/lifestyle advice, or an active pharmacological comparator (comparison), improve hepatic steatosis grade, total and LDL cholesterol, and serum alanine aminotransferase (ALT) and aspartate aminotransferase (AST) (primary outcomes)? Secondary outcomes were triglycerides, HDL cholesterol, alkaline phosphatase (ALP), gamma-glutamyl transferase (GGT), fasting blood sugar (FBS), systolic blood pressure (SBP), diastolic blood pressure (DBP), IL-1, IL-6, TNF-α, high-sensitivity C-reactive protein (hs-CRP), body weight, waist circumference, and adverse events.

## Materials and Methods

No patients were directly involved in the design, conduct, or reporting of this meta-analysis. This study utilized data from previously published controlled clinical trials. As the data were publicly available and no new patient recruitment or data collection was conducted, Institutional Review Board (IRB) approval was not required.

### Data sources and search strategy

In this study, the Preferred Reporting Items for Systematic Reviews and Meta-Analyses (PRISMA 2020) guidelines were followed [10]. A comprehensive literature search was conducted from database inception to December 2025 in PubMed/MEDLINE, Embase, the Cochrane Library (CENTRAL), and Web of Science. The full search string is reported in Supplementary Material 1 (jocmr.elmerjournals.com). We used a combination of several keywords and their corresponding subject term headings in our search, including, but not limited to, “Non-alcoholic Fatty Liver Disease” OR “MASLD,” OR “nonalcoholic steatohepatitis” OR “NASH” OR “fatty liver,” “hepatic fibrosis,” “steatohepatitis,” “melatonin,” MeSH terms, and Boolean operators “AND” and “OR.” In addition, the bibliographic sections of the selected articles from the online search were manually screened for any other relevant studies. No language or publication-date restrictions were applied. All eligible trials identified were published in English; no non-English records were retrieved. The results of the search are summarized in Figure 1.

**Figure 1:**
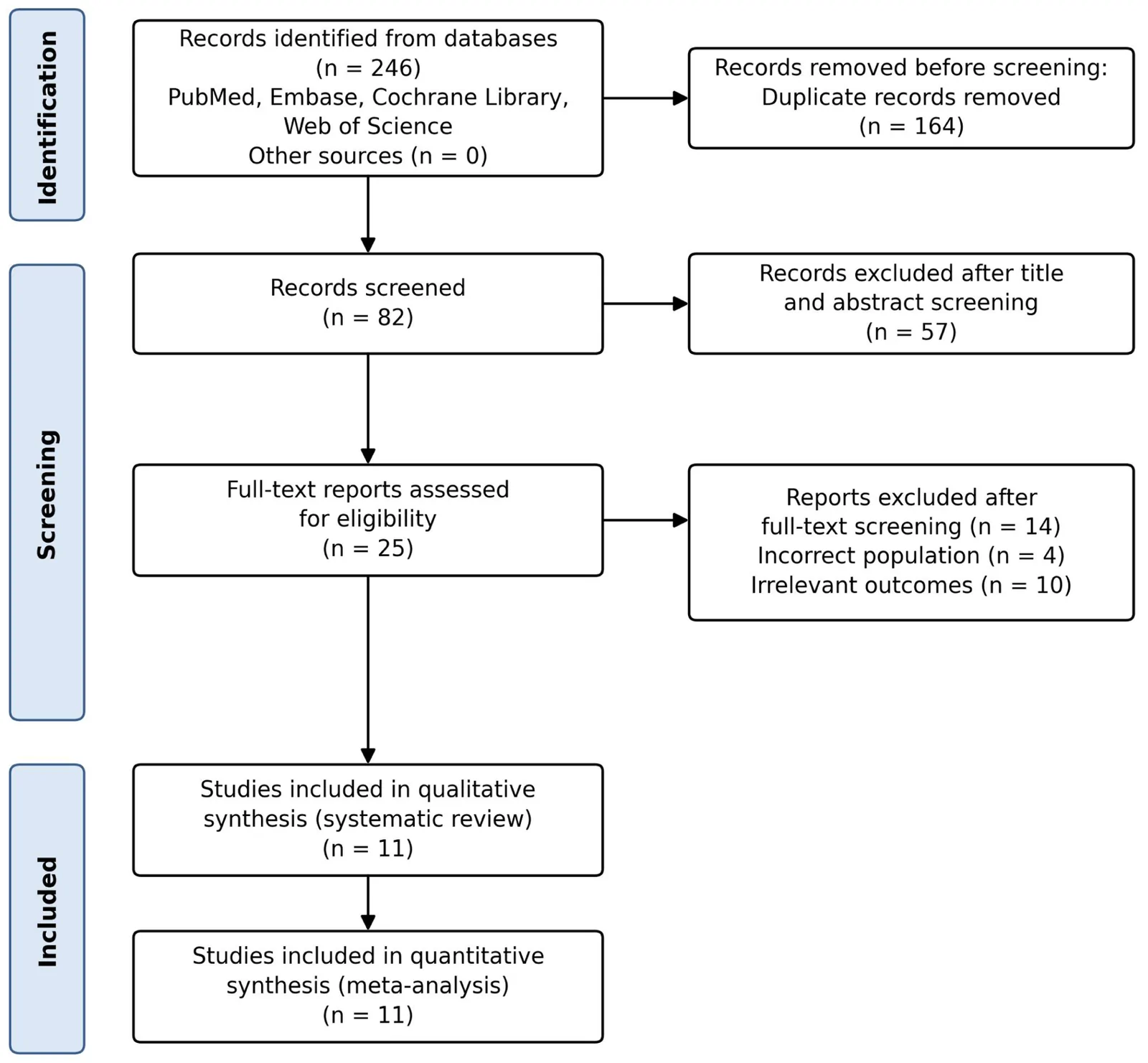
PRISMA 2020 flowchart summarizing results of literature search.

### Study selection and selection criteria

Studies were eligible if they (1) were controlled clinical trials; randomized (parallel-group or crossover) or non-randomized with a concurrent control group in adults (≥ 18 years) with MASLD/non-alcoholic fatty liver disease (NAFLD) diagnosed by imaging, histology, or clinical criteria; (2) compared melatonin supplementation of any dose, formulation, or duration, given alone or as add-on therapy, with placebo, no treatment or lifestyle advice, or an active pharmacological comparator; and (3) reported at least one of the prespecified outcomes. Because all trials addressed the effect of adding melatonin to the background management received by the control arm, trials with placebo, no-treatment, and active comparators were initially pooled; the influence of comparator type was to be examined in prespecified subgroup analyses, which ultimately could not be performed (see “Statistical analysis”). Single-arm studies, uncontrolled before–after studies, observational studies, case reports and series, editorials, reviews, animal studies, and records available only as conference abstracts or without full text were excluded. No restrictions were placed on language, publication date, or geographic setting.

### Outcomes

Primary outcomes were prespecified as (1) hepatic steatosis grade (I, II, III) at end of treatment, (2) total cholesterol, (3) LDL cholesterol, (4) ALT, and (5) AST. Secondary outcomes were triglycerides, HDL cholesterol, ALP, GGT, FBS, SBP, DBP, IL-1, IL-6, TNF-α, hs-CRP, body weight, waist circumference, and adverse events.

Hepatic steatosis grade was defined according to each trial’s ultrasonographic criteria as grade I (mild: minimally increased echogenicity with normal visualization of the diaphragm and intrahepatic vessel walls), grade II (moderate: moderately increased echogenicity with slightly impaired visualization) or grade III (severe: markedly increased echogenicity with poor or no visualization of the diaphragm, vessel walls, and posterior right lobe). Only trials using ultrasonographic grading were pooled for this outcome (n = 3); Bahrami et al [3], Pakravan et al [11], and Mohammadi et al [12] graded steatosis by ultrasonography and contributed to this outcome. Celinski et al [4], Cichoz-Lach et al [13], and the three Gonciarz et al reports [14–16] established the diagnosis by liver biopsy at baseline but did not grade steatosis at the end of treatment, Mojtahedi et al [17] graded steatosis by controlled attenuation parameter on transient elastography (S1–S3) rather than by ultrasonography, and Chojnacki et al [18] and Esmaeili et al [19] did not assess steatosis; these trials contributed only to the biochemical and anthropometric outcomes. Because assessment modalities otherwise varied across trials (imaging-based techniques and non-invasive clinical or biochemical parameters), this was recorded as a potential source of heterogeneity.

### Data extraction and quality assessment

Results were exported to EndNote 20 (Clarivate, Philadelphia, Pennsylvania, United States) and duplicates were removed by algorithmic deduplication as well as manually. Two authors (FH and SHQ) independently reviewed the title and abstracts of the identified studies and excluded any article that did not answer the research query or did not follow predefined selection criteria. The full text of the remaining articles was studied to see if they contained any relevant information. Inter-reviewer disagreements were resolved through discussion, and consensus was reached with the involvement of a third investigator (AS).

For continuous outcomes we extracted the mean and standard deviation (SD) at end of treatment for each arm. Where a trial reported standard errors, 95% confidence intervals (CIs) or interquartile ranges (IQRs) instead of SDs, these were converted according to the Cochrane Handbook (Section 6.5.2) and, for medians with IQRs, the method of Wan et al [20]. These conversions were required for three trials (Cichoz-Lach et al 2010, which reported standard errors, and Gonciarz et al 2010 and 2012, which reported medians and quartiles). Where units differed between trials (e.g., mmol/L vs. mg/dL), values were converted to a common unit before pooling. Adverse-event data (any adverse event, specific events, withdrawals due to adverse events, serious adverse events) were extracted as reported.

Risk of bias was assessed independently by two reviewers. The randomized trials were assessed with the revised Cochrane Risk-of-Bias 2.0 (RoB 2) tool for randomized trials, which addresses five domains: bias arising from the randomization process, bias due to deviations from intended interventions, bias due to missing outcome data, bias in measurement of the outcome, and bias in selection of the reported result; each trial was judged to be at low risk, of some concern, or at high risk of bias overall [21]. The non-randomized controlled trials were assessed with the Risk Of Bias In Non-randomized Studies of Interventions (ROBINS-I) tool, which covers confounding, selection of participants, classification of interventions, deviations from intended interventions, missing data, measurement of outcomes, and selection of the reported result, with overall judgements of low, moderate, serious, or critical risk of bias [22]. We also used Grading of Recommendations Assessment, Development and Evaluation (GRADE) assessment to evaluate outcomes in the study, which evaluate the strength of evidence on the following domains: (1) risk of bias, (2) inconsistency, (3) indirectness, (4) imprecision [23], and (5) publication bias, which was considered qualitatively because formal tests were not feasible (see “Statistical analysis”). Where non-randomized trials contributed to an outcome, this was reflected in the risk-of-bias domain by rating that domain as serious. GRADEPro GDT was employed to construct the summary of findings (SoF).

### Statistical analysis

All analyses were performed in Review Manager (RevMan) version 5.4 (The Cochrane Collaboration, 2020). Dichotomous outcomes (proportion of participants with grade I, II, or III steatosis at end of treatment) were pooled as risk ratios (RRs) with 95% CIs using the Mantel–Haenszel method. Continuous outcomes were pooled as mean differences (MDs) with 95% CI using the inverse-variance method. Random-effects (DerSimonian–Laird) models were used for all outcomes because clinical and methodological heterogeneity was anticipated. A P-value < 0.05 was considered statistically significant. Forest plots were created to visualize the results. Heterogeneity across studies was calculated using the I^2^ statistics, with values of 0%, 25%, 50%, and 75% indicating absent, low, moderate, and high heterogeneity, respectively [24]. Sensitivity analyses were undertaken for outcomes with substantial heterogeneity (I^2^ ≥ 50%). Trials were omitted, one or more at a time, if (1) their CI did not overlap the pooled estimate on visual inspection of the forest plot, or (2) they differed materially in design from the remaining trials (co-administration of essential phospholipids in both arms, an active or non-placebo comparator, a statin-treated population, or a crossover design). The reason for each omission is listed in Supplementary Material 2 (jocmr.elmerjournals.com). These analyses are exploratory and were not used to draw efficacy conclusions. Subgroup analyses by comparator type (placebo or no treatment vs. active pharmacological comparator), melatonin daily dose (≤ 6 mg vs. ≥ 10 mg), treatment duration (< 12 weeks vs. ≥ 12 weeks), and study design (randomized vs. non-randomized) were prespecified but could not be performed because of the heterogeneity between trials and the small number of trials per outcome (see “Results”). Funnel plots and statistical tests for funnel-plot asymmetry were not performed because fewer than 10 trials contributed to every outcome, and such tests lack power below this number; the possibility of publication bias was instead considered qualitatively when rating the certainty of evidence with GRADE (domain “other considerations”), and the certainty for ALT and AST was downgraded on this basis (Supplementary Material 3, jocmr.elmerjournals.com).

## Results

### Literature search results

The search of PubMed, Embase, the Cochrane Library, and Web of Science retrieved 246, of which 82 studies were left after removing duplicates. Fifty-seven articles were excluded after the title and abstract screening. After screening for full text, 14 more articles were excluded which did not meet the inclusion criteria. Therefore, a total of 11 clinical trials were included in the meta-analysis [3, 4, 11–19]. The PRISMA flow chart (Fig. 1) summarizes the results of our literature search. Nine trials were placebo-controlled (in three of them essential phospholipids or statin therapy was given in both arms); Celinski et al [4] used essential phospholipids alone as the control, and Gonciarz et al 2013 [16] compared treated patients with an untreated healthy reference group. No trial used an active pharmacological comparator.

### Study characteristics and quality assessment

Eleven trials met the inclusion criteria, comprising nine randomized controlled trials [3, 4, 11, 12, 14, 15, 17–19] and two non-randomized controlled trials [13, 16], with 548 participants. The melatonin intervention group included 300 patients, while the control group had 248 patients. The overall mean age was 41.57 ± 9.10 years. The baseline characteristics of the included studies are summarized in Table 1.

**Table 1:** Baseline Characteristics of Intervention and Control Groups in the Included Studies

Author, year (Ref)	Design	Population/diagnosis of steatosis	Number of analyzed (Mel/Ctrl)	Mean age, years (Mel/Ctrl)	Male, n (%) (Mel/Ctrl)	Melatonin regimen	Comparator (type)	Duration	Weight, kg (Mel/Ctrl)	BMI, kg/m^2^ (Mel/Ctrl)
Bahrami, 2020 [3]	RCT, double-blind, parallel	NAFLD; ultrasound (steatosis ≥ grade 1) + ALT > 30 (M)/> 19 (F) U/L	24/21	44.0 ± 9.62/37.71 ± 11.31	17 (71)/14 (67)	6 mg (2 × 3 mg) orally, 1 h before bedtime	Starch placebo, matched shape/color (placebo)	12 weeks	85.45 ± 11.4/95.47 ± 16.2	29.46 ± 3.6/32.59 ± 6.1
Celinski, 2014 [4]	RCT, open-label, three-arm (melatonin and Essentiale-only arms used)	NAFLD confirmed by liver biopsy (simple steatosis 17, NASH 6 per arm)	23/23	36.16 ± 5.77/29.33 ± 9.58	8 (35)/12 (52)	2 × 5 mg/day orally + Essentiale forte 3 × 300 mg/day	Essentiale forte alone, no placebo (no melatonin)	14 months	NR/NR	NR/NR
Esmaeili, 2021 [19]	RCT, double-blind, two-period crossover	Chronic liver disease with pruritus (PSC, PBC, cirrhosis, AIH, HBV/HCV); steatosis not assessed	40 (crossover; 18 melatonin-first/22 placebo-first)	41.5 ± 12.08/49.73 ± 12.89 (by sequence)	11 (61)/11 (50) (by sequence)	10 mg (2 × 5 mg pearls) at night	Placebo pearls of identical shape, size, color and odor (placebo)	2 weeks per period, 2-week washout	NR/NR	NR/NR
Cichoz-Lach, 2010 [13]	Non-randomized controlled trial (allocation not described), three-arm	NASH confirmed by liver biopsy	15/15	NR/NR (all patients: women 22–46 years, men 26–58 years)	NR/NR (27/45 men overall)	2 × 5 mg/day orally + Essentiale forte 3 × daily	Placebo + Essentiale forte (placebo); (tryptophan arm not used)	4 weeks	NR/NR	NR/NR
Gonciarz, 2010 [14]	RCT (2:1 random allocation reported in the 2012 extension paper), placebo-controlled	NASH confirmed by liver biopsy; ALT/AST elevated	30/12	41.5 ± 4.0/40.8 ± 3.6 (from Ref [15])	18 (60)/8 (67) (from ref 15)	2 × 5 mg/day orally (09:00 and 21:00) + diet and walking program	Placebo twice daily + same lifestyle program (placebo)	12 weeks	82.5 ± 3.4/81.1 ± 3.7 (from ref 15)	NR/NR
Gonciarz, 2012 [15]	RCT (2:1 random allocation), placebo-controlled; extension of ref 14 in the same 42 patients	NASH confirmed by liver biopsy	30/12	41.5 ± 4.0/40.8 ± 3.6	18 (60)/8 (67)	2 × 5 mg/day orally (09:00 and 21:00) + diet and walking program	Placebo twice daily + same lifestyle program (placebo)	24 weeks (+ 12-week off-treatment follow-up)	82.5 ± 3.4/81.1 ± 3.7	NR/NR
Pakravan, 2017 [11]	RCT, double-blind, parallel	NAFLD by clinical and pathological findings; ultrasound grading (mild/moderate/severe)	49/48	42.5 ± 10.1/40.6 ± 9.8	33 (69)/27 (56)	One tablet orally twice daily (dose in mg not stated; abstract states “thrice daily for 3 months”)	Placebo tablets manufactured identically (placebo)	6 weeks (abstract states 3 months)	85.1 ± 13.5/86.9 ± 13.7	NR/NR
Chojnacki, 2017 [18]	RCT (allocation method not described), placebo-controlled	Hyperlipidemia on statins with ≥ 2-fold elevated aminotransferases; BMI > 30 excluded; steatosis not assessed	30/30	NR/NR (all patients 47–65 years)	NR/NR (19/60 men overall)	2 × 5 mg/day orally (07:00 and 21:00) + statin 20 mg/day + 1,600-kcal diet	Placebo at the same dose and times + same statin and diet (placebo)	6 months	NR / NR	NR/NR (≤ 30 by design)
Mohammadi, 2022 [12]	RCT, double-blind, four-arm (melatonin and placebo arms used)	NAFLD by ultrasonography + elevated ALT/AST; diabetes excluded	35/35	47.5 ± 13.02/44.4 ± 2.07	16 (46)/22 (63)	10 mg once daily at night + two placebos + same diet and activity program	Three placebos daily + same diet and activity program (placebo)	24 weeks	74.89 ± 11.88/89.44 ± 11.23	27.6 ± 3.8/28.05 ± 4.27
Mojtahedi, 2023 [17]	RCT, double-blind, four-arm (melatonin and placebo arms used)	NAFLD by transient elastography (FibroScan) with CAP steatosis S1–S3 and elevated ALT; diabetes and metabolic syndrome excluded	30/30	43.66 ± 11.01/43.12 ± 10.87	12 (40)/12 (40)	10 mg once daily orally + 500-kcal-deficit weight-loss diet	Placebo, pre-packaged and encoded, indistinguishable + same diet (placebo)	3 months	88.54 ± 15.06/93.05 ± 10.33	31.39 ± 2.82/32.57 ± 2.55
Gonciarz, 2013 [16]	Non-randomized before–after study in NASH patients with an untreated lean healthy reference group (no MASLD control arm)	NASH by liver biopsy within 12 months, overweight (BMI 25–30), aminotransferases/GGT > 1.5 × ULN; reference group: 15 lean healthy subjects	16/15 (healthy, untreated)	42.1 ± 3.4/40.5 ± 3.8	11 (69)/7 (47)	2 × 5 mg/day orally (09:00 and 21:00)	Untreated healthy subjects (no treatment; not a MASLD control arm)	28 days	NR/NR	28.6 ± 1.9/23.1 ± 1.1

Values are mean ± SD unless stated. AIH: autoimmune hepatitis; ALT: alanine aminotransferase; AST: aspartate aminotransferase; BMI: body mass index; CAP: controlled attenuation parameter; Ctrl: control arm; GGT: gamma-glutamyl transferase; HBV: hepatitis B virus; HCV: hepatitis C virus; Mel: melatonin arm; NAFLD: non-alcoholic fatty liver disease; NASH: non-alcoholic steatohepatitis; NR: not reported; PBC/PSC: primary biliary/sclerosing cholangitis; RCT: randomized controlled trial; ULN: upper limit of normal.

The revised Cochrane RoB 2 tool was used to assess the quality and risk of bias of the randomized trials (Supplementary Material 4a, jocmr.elmerjournals.com), and ROBINS-I was used for the non-randomized trials (Supplementary Material 4b, jocmr.elmerjournals.com). Of the nine randomized trials, one was judged at low risk of bias overall (Mojtahedi 2023) [17], six had some concerns (Bahrami 2020 [3], Esmaeili 2021 [19], Gonciarz 2010 [14], Gonciarz 2012 [15], Chojnacki 2017 [18], and Mohammadi 2022 [12]), most often because the method of sequence generation or allocation concealment was not described, and two were at high risk (Celinski 2014 [4], because of an undescribed randomization procedure with substantial baseline imbalance and no blinding; Pakravan 2017 [11], because of internally inconsistent reporting of the regimen, denominators, and adverse events). Of the two non-randomized studies, Cichoz-Lach 2010 [13] was at serious risk of bias (uncontrolled confounding) and Gonciarz 2013 [16] at critical risk (comparison of treated patients with an untreated healthy reference group). Domain-level judgements with supporting text are given in Supplementary Materials 5 and 6 (jocmr.elmerjournals.com). Additionally, GRADE SoF is given in Supplementary Material 3 (jocmr.elmerjournals.com).

### Primary outcomes

#### Grades of steatosis

There was no significant difference in the proportion of participants with grade I (RR = 0.86, 95% CI: 0.39–1.92, P = 0.72, I^2^ = 71%; three trials) or grade II steatosis (RR = 0.73, 95% CI: 0.44–1.21, P = 0.22, I^2^ = 0%; three trials) at end of treatment. The proportion of participants classified as grade III steatosis at end of treatment was lower in the melatonin group (RR = 0.23, 95% CI: 0.07–0.77, P = 0.02, I^2^ = 0%; two trials, 126 participants, 16 events) (Fig. 2). Because this outcome describes the distribution of grades at follow-up rather than within-participant change, and is based on few events, it should not be interpreted as evidence that melatonin reverses severe steatosis.

**Figure 2:**
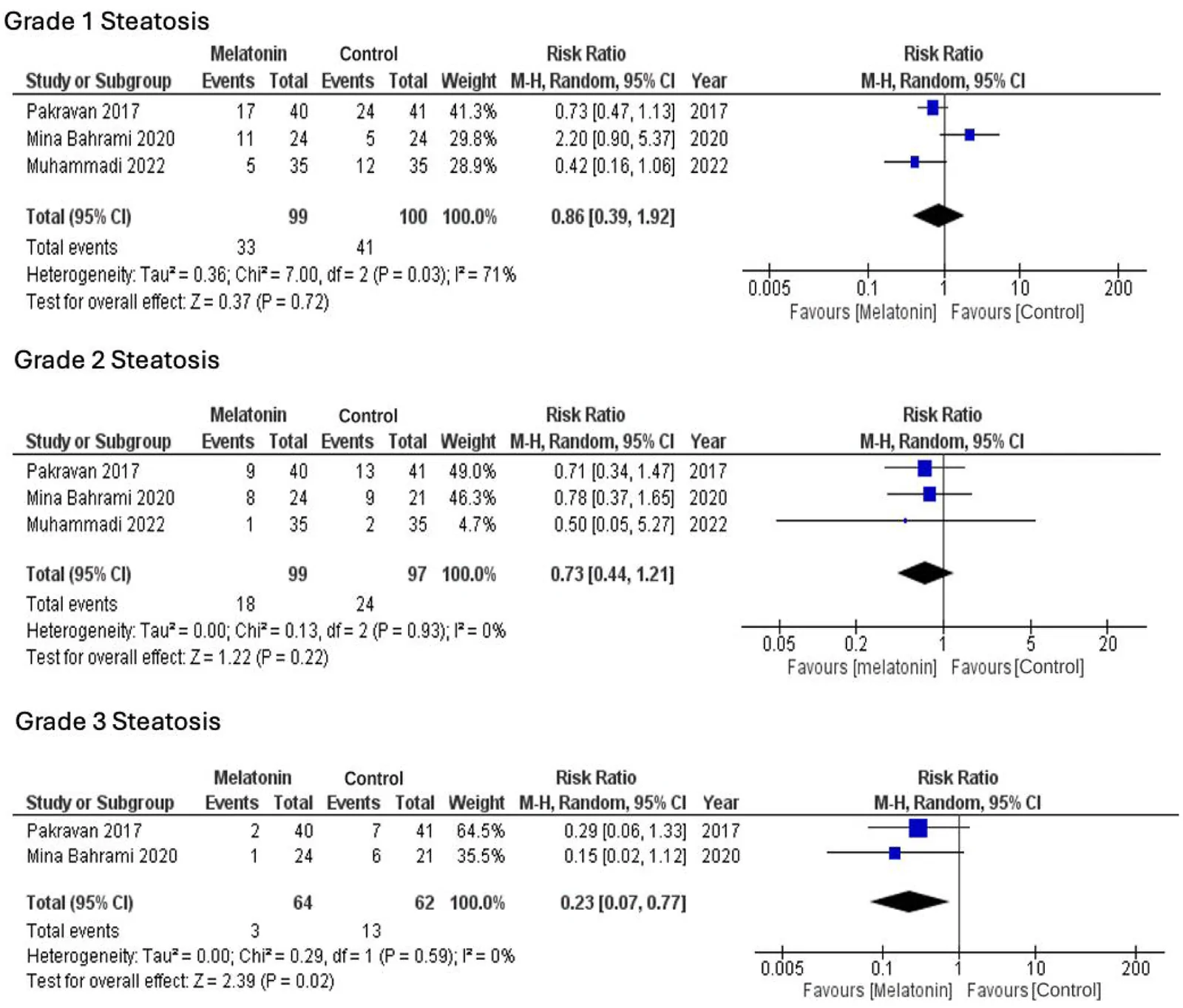
Forest plot comparing grades of steatosis between melatonin and control groups.

#### Cholesterol and LDL

The pooled analysis showed a statistically significant reduction in total cholesterol (MD: −16.80 mg/dL, 95% CI: −29.13 to −4.47, P = 0.008, I^2^ = 76%; seven trials) and LDL (MD: −52.4 mg/dL, 95% CI: −100.52 to −4.27, P = 0.03, I^2^ = 96%; five trials) in the melatonin group (Fig. 3).

**Figure 3:**
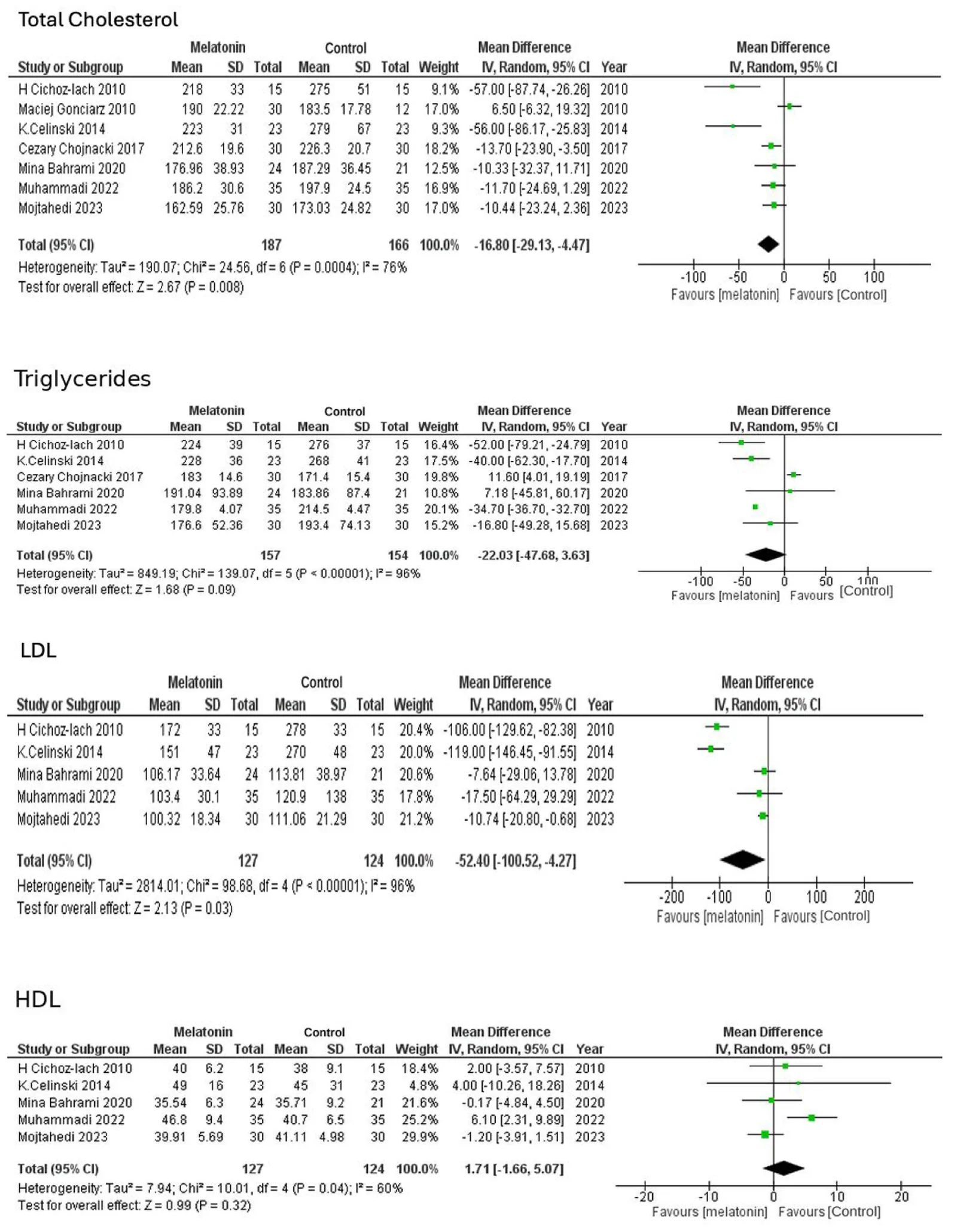
Forest plot comparing total cholesterol, LDL, HDL, and triglycerides between the melatonin and control groups. HDL: high-density lipoprotein; LDL: low-density lipoprotein.

#### ALT and AST

The analysis reported no significant difference in mean ALT (MD: −7.97 IU/L, 95% CI: −17.43 to 1.49, P = 0.10, I^2^ = 84%; 10 trials) and AST value (MD: −5.23 IU/L, 95% CI: −14.12 to 3.67, P = 0.25, I^2^ = 91%; 10 trials) between the melatonin and control group (Fig. 4).

**Figure 4:**
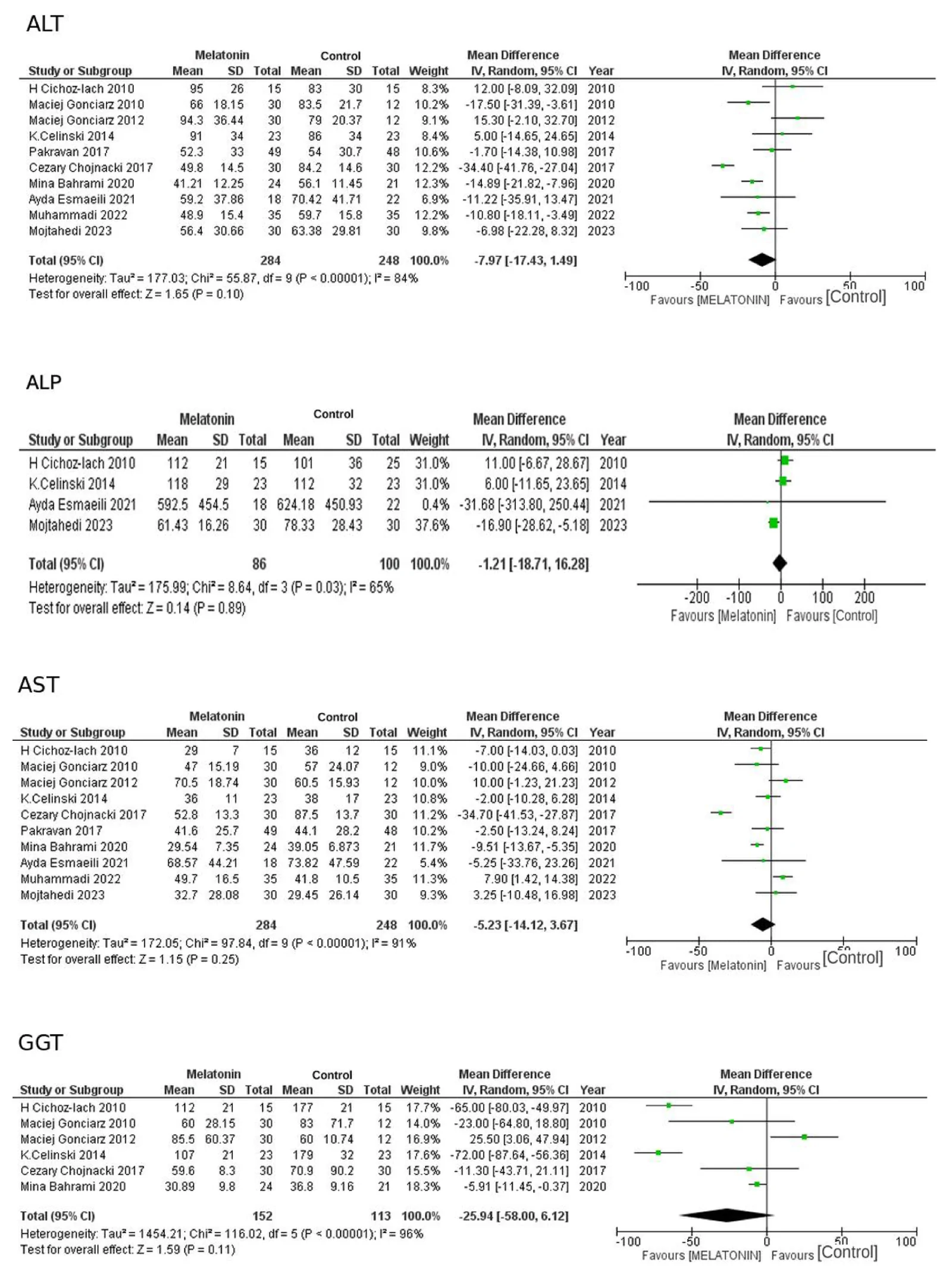
Forest plot showing changes in liver enzymes including ALT, AST, GGT, and ALP between melatonin and control groups. ALP: alkaline phosphatase; ALT: alanine aminotransferase; AST: aspartate aminotransferase; GGT: gamma-glutamyl transferase.

### Secondary outcomes

#### Triglycerides and HDL

There was no significant reduction in triglycerides (MD: −22.03 mg/dL, 95% CI: −47.68 to 3.63, P = 0.09, I^2^ = 96%; six trials) and HDL levels (MD: 1.71 mg/dL, 95% CI: −1.66 to 5.07, P = 0.32, I^2^ = 60%; five trials) in the melatonin group (Fig. 3).

#### ALP

There was no significant reduction in serum ALP levels (MD: −1.21 IU/L, 95% CI: −18.71 to 16.28, P = 0.89, I^2^ = 65%; four trials) (Fig. 4).

#### GGT

There was no significant decrease in serum GGT levels upon treatment with melatonin (MD: −25.94 IU/L, 95% CI: −58.00 to 6.12, P = 0.11, I^2^ = 96%; six trials) (Fig. 4).

#### FBS

There was no significant decrease in the mean FBS levels among patients treated with melatonin (MD: −5.98 mg/dL, 95% CI: −13.30 to 1.34, P = 0.11, I^2^ = 52%; three trials) (Fig. 5).

**Figure 5:**
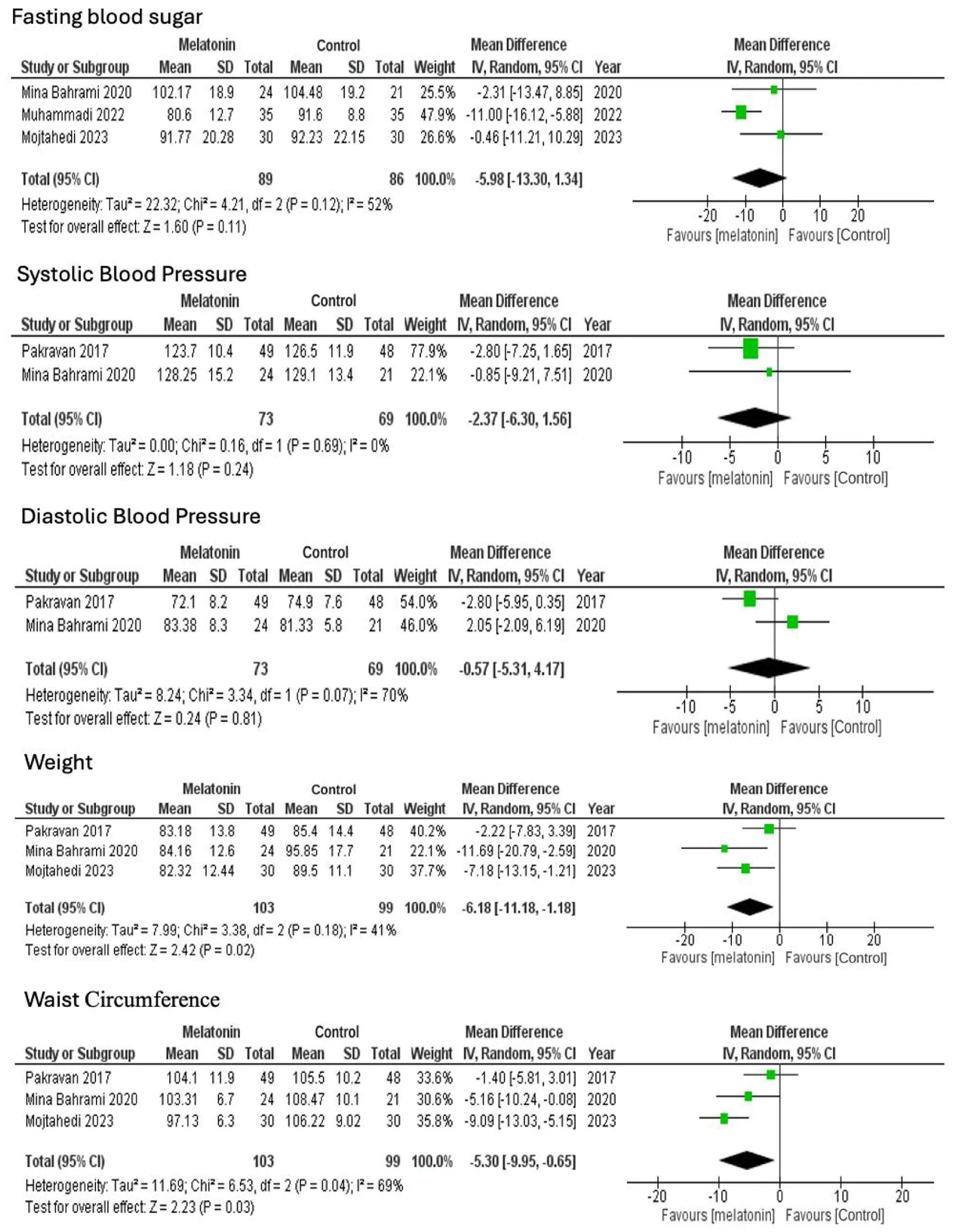
Forest plot comparing FBS, BP, weight, and waist circumference between melatonin and control groups. BP: blood pressure; FBS: fast blood sugar.

#### SBP and DBP

The pooled estimates demonstrated an insignificant decrease in the SBP (MD: −2.37 mm Hg, 95% CI: −6.30 to 1.56, P = 0.24, I^2^ = 0%) and DBP (MD: −0.57 mm Hg, 95% CI: −5.31 to 4.17, P = 0.81, I^2^ = 70%) values (Fig. 5).

#### IL-1, IL-6, TNF-α, and hs-CRP

There was a significant reduction in IL-1 (MD: −4.40 pg/mL, 95% CI: −5.55 to −3.26, P < 0.00001, I^2^ = 9%; two trials), IL-6 (MD: −4.28 pg/mL, 95% CI: −6.80 to −1.76, P = 0.0009, I^2^ = 0%; two trials), and TNF-α (MD: −0.48 pg/mL, 95% CI: −0.88 to −0.09, P = 0.02, I^2^ = 80%; three trials) in the melatonin group (Fig. 6).

**Figure 6:**
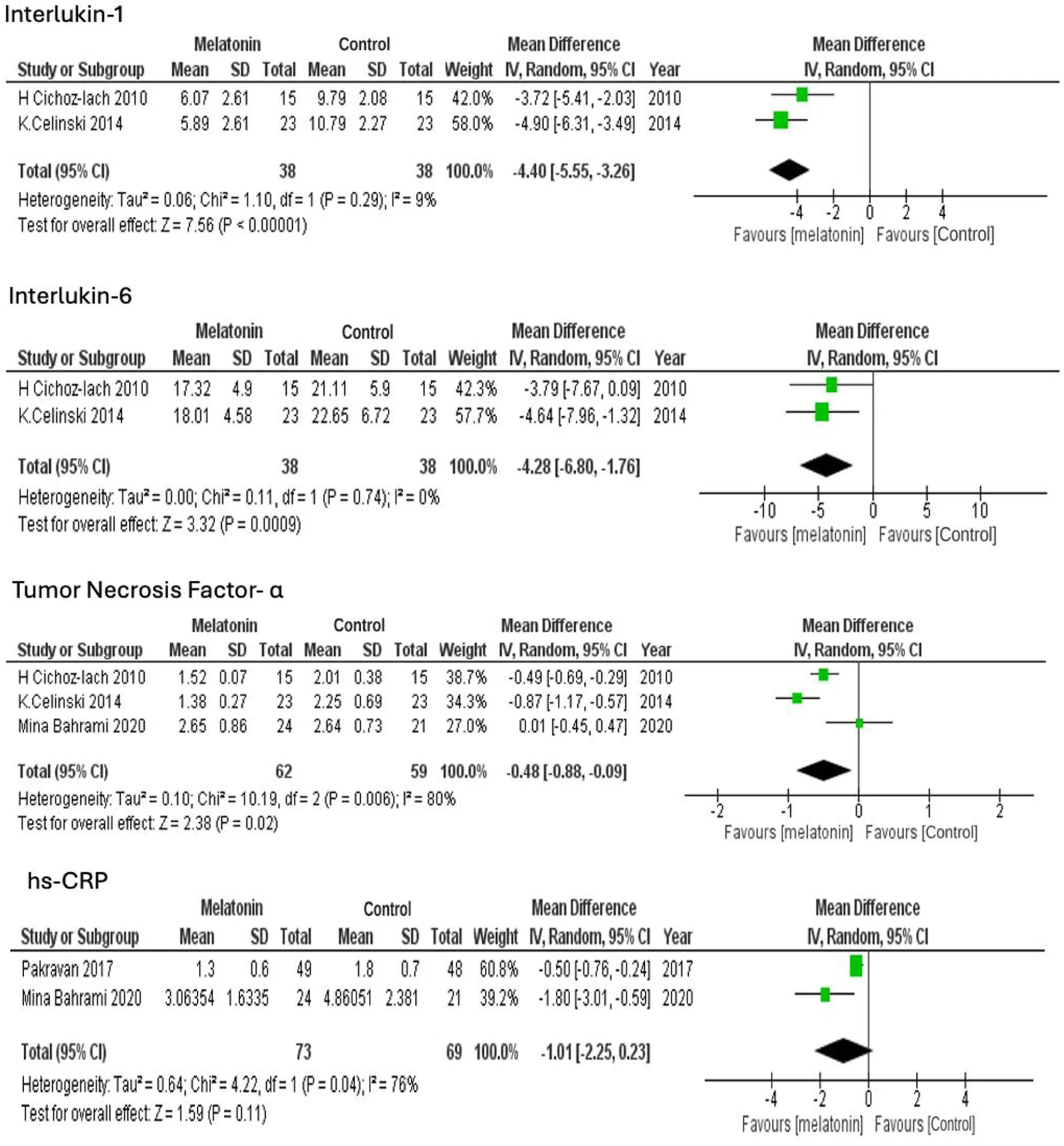
Forest plot comparing changes in inflammatory markers between melatonin and control groups.

Melatonin supplementation showed no significant decrease in hs-CRP levels (MD: −1.01 ng/mL, 95% CI: −2.25 to 0.23, P = 0.11, I^2^ = 76%; two trials) (Fig. 6).

#### Weight and waist circumference

There was a significant decrease in weight (MD: −6.18 kg (95% CI: −11.18 to −1.18, P = 0.02, I^2^ = 41%) and waist circumference (MD: −5.30 cm (95% CI: −9.95 to −0.65, P = 0.03, I^2^ = 69%) in the melatonin intervention group (Fig. 5).

### Subgroup analyses

The prespecified subgroup analyses (by comparator type, dose, duration, and study design) could not be performed because of the heterogeneity between trials: comparators, populations, background co-interventions, treatment durations, and outcome-assessment methods varied widely, and few trials contributed to each outcome, so that meaningful subgroups could not be formed. The influence of individual trials was instead explored in the exploratory sensitivity analyses described below.

### Exploratory sensitivity analyses

For outcomes with I^2^ ≥ 50%, we examined the influence of individual trials by omitting those whose CIs did not overlap the pooled estimate and/or that differed in design (add-on essential phospholipids in both arms, active or non-placebo comparator, statin-treated population, crossover design). These analyses were not powered hypothesis tests and are reported to characterize heterogeneity, not to establish efficacy (Supplementary Materials 2 and 7–16, jocmr.elmerjournals.com). For grade I steatosis, omission of Bahrami et al (2020) [3] did not change the conclusion (RR = 0.64, 95% CI: 0.40–1.02, P = 0.06, I^2^ = 15%) (Supplementary Material 7, jocmr.elmerjournals.com). For total cholesterol, omission of Celinski et al (2014), Cichoz-Lach et al (2010), and Gonciarz et al (2010) [4, 13, 14] reduced heterogeneity without changing the conclusion (MD: −12.07 mg/dL, 95% CI: −18.56 to −5.57, P = 0.0003, I^2^ = 0%) (Supplementary Material 8, jocmr.elmerjournals.com); for LDL, omission of Celinski et al (2014) and Cichoz-Lach et al (2010) [4, 13] gave MD: −10.45 mg/dL (95% CI: −19.38 to −1.51, P = 0.02, I^2^ = 0%) (Supplementary Material 9, jocmr.elmerjournals.com). After exclusion of two to three trials, the point estimates for ALT (omitting Cichoz-Lach et al 2010, Gonciarz et al 2012, and Pakravan et al 2017 [11, 13, 15]: MD: −10.53 IU/L, 95% CI: −15.19 to −5.87, I^2^ = 13%; Supplementary Material 10, jocmr.elmerjournals.com), AST (omitting Gonciarz et al 2012, Chojnacki et al 2017, and Mohammadi et al 2022 [12, 15, 18]: MD: −6.92 IU/L, 95% CI: −9.90 to −3.94, I^2^ = 0%; Supplementary Material 11, jocmr.elmerjournals.com), GGT (omitting Celinski et al 2014, Cichoz-Lach et al 2010, and Gonciarz et al 2012 [4, 13, 15]: MD: −6.35 IU/L, 95% CI: −11.76 to −0.93, I^2^ = 0%; Supplementary Material 12, jocmr.elmerjournals.com) and triglycerides (omitting Chojnacki et al 2017 [18]: MD: −34.58 mg/dL, 95% CI: −44.30 to −24.85, I^2^ = 25%; Supplementary Material 13, jocmr.elmerjournals.com) reached nominal significance; because the direction and size of these estimates depended on which trials were removed, they are hypothesis-generating only. Omission of Mohammadi et al (2022) [12] for HDL (MD: −0.40 mg/dL, 95% CI: −2.53 to 1.74, I^2^ = 0%; Supplementary Material 14, jocmr.elmerjournals.com) and for FBS (MD: −1.35 mg/dL, 95% CI: −9.09 to 6.39, I^2^ = 0%; Supplementary Material 15, jocmr.elmerjournals.com), and of Mojtahedi et al (2023) [17] for ALP (MD: 8.42 IU/L, 95% CI: −4.05 to 20.89, I^2^ = 0%; Supplementary Material 16, jocmr.elmerjournals.com), removed heterogeneity without altering the non-significant conclusions.

### Adverse events

Nine of the 11 trials reported on adverse events [3, 4, 11, 14–19], and two of them only stated that no serious adverse effects occurred [11, 17]; one trial described adverse-event monitoring but reported no results [12], and one did not report adverse events [13] (Supplementary Material 17, jocmr.elmerjournals.com). No serious adverse event attributed to melatonin was reported in any trial, and no trial reported adverse events systematically by arm with denominators. Bahrami et al (6 mg/day for 12 weeks) [3], Celinski et al (10 mg/day for 14 months) [4], and Gonciarz et al 2013 (10 mg/day for 28 days) [16] reported no adverse effects; Pakravan et al [11] and Mojtahedi et al [17] reported no serious side effects (Pakravan et al describing the rates of other side effects as similar between groups, data not shown). The most frequently reported effect was somnolence or fatigue: slight somnolence in 12 of 30 (40%) melatonin-treated participants during the first 4 weeks in Gonciarz et al 2010 [14], none during the subsequent 12 weeks of the extension study [15], and mild morning fatigue in six of 30 (20%) melatonin-treated participants during the first 2 weeks in Chojnacki et al [18] (0 of 30 with placebo). In the crossover trial of Esmaeili et al (10 mg/day) [19], four of 49 enrolled participants discontinued melatonin because of adverse drug reactions (drowsiness, n = 2; headache, n = 1; gastrointestinal disturbance, n = 1; all rated “probable” on the Naranjo scale) and two discontinued placebo because of gastrointestinal upset (five placebo-phase complaints in total); these were the only withdrawals attributed to adverse effects in any trial. Adverse-event data were too sparse and inconsistently reported to be pooled.

## Discussion

This systematic review and meta-analysis synthesized data from 11 controlled clinical trials (nine randomized and two non-randomized) and included 548 participants aiming to assess the efficacy of melatonin supplementation as a treatment modality in the management of MASLD. Analysis revealed improvements in several metabolic, inflammatory, and anthropometric outcomes in patients with MASLD, although some findings, particularly liver enzyme reductions, were significant only after sensitivity analyses and should be considered exploratory. Fewer melatonin-treated participants were classified as grade III steatosis at follow-up in two small trials, but grade I and II steatosis did not differ, so the steatosis findings are inconsistent and do not establish regression of steatosis.

The pathogenetic mechanisms of MASLD are multifactorial. Ghosh et al comprehensively elucidated the dynamic interplay of high-fat diet, adipose tissue dysregulation, *de-novo* lipid synthesis, and hepatic steatosis as primary drivers of the metabolic dysfunction first seen in MASLD [25]. Atherogenic dyslipidemia, characterized by hypertriglyceridemia, high LDL-C, and low HDL-C levels, is a well-documented risk factor for cardiovascular diseases which is the leading cause of death in patients with MASLD [26]. Several animal and human studies have highlighted the hypolipidemic effect of melatonin on subjects with metabolic impairments [8]. Our study results are consistent with antecedent evidence revealing the anti-hyperlipidemic action of melatonin by modulation of lipid synthesis pathways, thereby, reducing serum cholesterol, LDL, and triglyceride levels [9]. Experimental studies suggest that melatonin may influence lipid metabolism through reduced intestinal cholesterol absorption, modulation of cholesterol synthesis and LDL receptor activity, effects on body weight, intraabdominal adiposity, and energy regulation [27–29]. However, these studies do not directly support decreased visceral adipose tissue lipolysis as a mechanism.

Although melatonin was associated with reductions in total cholesterol, LDL, and body weight, the clinical magnitude of these changes should be interpreted in context. The observed reductions in total cholesterol (about 17 mg/dL), LDL cholesterol (about 10 mg/dL after exclusion of two outlying trials), and body weight (about 6 kg, from two trials) are modest in absolute terms and, for the anthropometric outcomes, rest on very few trials. No included trial compared melatonin head-to-head with statins, glucagon-like peptide 1 (GLP-1) receptor agonists, pioglitazone, or resmetirom, so no conclusion about relative efficacy can be drawn from these data. Melatonin should therefore be regarded, at most, as a potential adjunct to—not a substitute for—guideline-supported lifestyle, cardiometabolic, and pharmacological management, pending direct comparative evidence.

Intrahepatic fat accumulation, followed by oxidative stress and mitochondrial dysfunction, promotes the production of reactive oxygen species (ROS), resulting in lipid peroxidation and excessive proinflammatory cytokines, a key pathogenic pathway in MASLD [30]. Cytokines play an important role in the progression of steatosis to inflammation and fibrosis seen in MASLD patients and amelioration of proinflammatory markers such as IL-1, Il-6, and TNF-α remains a mainstay in the management of liver fibrosis [5]. Melatonin exerts its cytoprotective effect by enhancing the *de-novo* synthesis of GSH and stimulating GPx, thus reducing injury and improving overall cell survival [5]. Apart from its chronobiotic effect, melatonin functions as a powerful free-radical scavenger and endogenous antioxidant reducing inflammation [6]. Our results corroborate previous findings of melatonin supplementation proving to be highly effective in reducing proinflammatory cytokine levels [4]. The reductions in IL-1, IL-6, and TNF-α observed here are consistent with melatonin’s antioxidant and anti-inflammatory actions; however, because this meta-analysis assessed circulating cytokines rather than histological, elastographic, or clinical endpoints, whether these biochemical changes translate into slower progression of liver injury remains to be established.

Serum aminotransferases and GGT are widely used markers of hepatocellular injury in MASLD; ALT and AST are, however, non-specific and correlate imperfectly with histological activity, while GGT has been proposed as a surrogate associated with disease progression and all-cause mortality [31, 32]. In our primary analyses, melatonin was not associated with statistically significant reductions in ALT (P = 0.10), AST (P = 0.25), or GGT (P = 0.11), and heterogeneity for these outcomes was high (I^2^ = 84–96%). Point estimates reached nominal significance only in exploratory sensitivity analyses after two or three trials had been excluded; because the direction and size of the effect depended on which trials were removed, these results are hypothesis-generating and do not establish an effect on liver enzymes. Although individual trials such as that of Gonciarz et al [16] reported enzyme reductions with melatonin, the pooled evidence is inconclusive. Moreover, a change in enzyme levels should not be equated with hepatoprotection, which would require histological, elastographic, or imaging endpoints that only one trial reported [17].

Previously, several studies have suggested that lifestyle modification with weight loss of about 5–10% could improve MASLD without any medical intervention emphasizing the importance of weight in this disease process [33]. Previous studies have shown discrepancies in the overall effect of melatonin supplementation on the improvement of anthropometric measures. Our findings were congruent with established evidence, as by Bahrami et al and Pakravan et al [3, 11], according to which melatonin significantly attenuates weight and waist circumference in patients with MASLD. The observed improvements in anthropometric measures may also be partially explained by melatonin’s metabolic effects, including its role in enhancing lipolytic activity and promoting lipid mobilization in adipose tissue, as discussed earlier, in addition to its circadian modulation of appetite, sleep, and gut microbiota composition [34].

Heterogeneity was substantial for several outcomes (I^2^ = 84–96% for ALT, AST, GGT, LDL cholesterol, and triglycerides), and several sources are identifiable. First, the comparator differed; nine trials used a placebo (three of them with essential phospholipids or a statin given in both arms), one used essential phospholipids without placebo [4], and one compared treated patients with an untreated healthy reference group [16]; no trial used an active pharmacological comparator. Although melatonin was thus always compared with “no melatonin,” background co-interventions (essential phospholipids, statin, structured diet, and exercise) that themselves lower liver enzymes or lipids differed between trials and are expected to dilute or inflate the observed contrast. Second, two trials were non-randomized, and their estimates are more susceptible to confounding and selection bias. Third, melatonin dose ranged from 6 to 10 mg/day (not stated in one trial [11]) and treatment duration from 2 weeks to 14 months, and two trials co-administered essential phospholipids in both arms. Fourth, steatosis was assessed with different modalities, and baseline enzyme and lipid concentrations differed across populations. Pooled estimates, particularly for lipids and liver enzymes, should therefore be regarded as approximate, and the direction of effect is more credible than its magnitude.

Adverse-event reporting in the included trials was incomplete: nine trials reported on adverse events, but two of these stated only that no serious adverse effects occurred, and none reported adverse events systematically by arm. The events described transient somnolence or morning fatigue in 20–40% of melatonin recipients in two trials, isolated headache and gastrointestinal upset were mild, and withdrawals attributable to melatonin occurred only in the crossover trial (four of 49 participants). In the broader literature, melatonin at doses similar to those used here (6–10 mg/day) is generally well tolerated; the most commonly reported effects are daytime somnolence, headache, dizziness, and nausea, and serious adverse events have not been consistently attributed to short-term use [35, 36]. Nevertheless, data on long-term use, drug interactions (including with anticoagulants, antihypertensives, and hypoglycemic agents), and use in patients with advanced liver disease in whom hepatic clearance of melatonin may be reduced remain limited. Systematic adverse-event monitoring should be a required component of future trials before melatonin can be recommended as an adjunctive therapy.

This meta-analysis has several strengths. To the best of our knowledge, our study is the first to account for various markers, including lipid profile, liver and metabolic markers, inflammatory markers, and anthropometric measures in patients with MASLD, offering a panoramic view of melatonin’s therapeutic potential. Nine of the included trials were randomized (eight of them placebo-controlled); the remaining two were non-randomized studies and were appraised separately with ROBINS-I. Sensitivity analyses were performed to explore sources of heterogeneity; however, because several estimates changed materially when individual trials were removed, these analyses highlight the fragility rather than the robustness of the enzyme and triglyceride findings.

From a clinical perspective, melatonin may be most beneficial as an adjunct therapy in patients with early MASLD or metabolic dysfunction who are not candidates for more aggressive pharmacologic interventions. Its low cost and wide availability would make melatonin attractive as an adjunct if efficacy and safety are confirmed, but the present evidence does not support its use in place of established therapies.

However, this systematic review and meta-analysis has several limitations. The number of eligible studies was small, and most included trials had limited sample sizes. Two studies were non-randomized, and none of the randomized trials was at low risk of bias overall, mainly because allocation concealment and blinding were incompletely described; two randomized trials were at high risk of bias. Two included trials enrolled populations that were not restricted to MASLD (chronic liver disease with pruritus [19] and statin-treated hyperlipidemia [18]), and two reports described the same randomized cohort at different time points [14, 15]. Incomplete reporting across studies meant that not all outcomes were consistently available, which may reduce the reliability of pooled estimates and introduce bias. The sources of heterogeneity discussed above further limit the generalizability of the pooled estimates. Publication bias could not be formally assessed because fewer than 10 trials contributed to any outcome, so funnel-plot asymmetry tests would have been underpowered; small-study effects therefore cannot be excluded, and the certainty of evidence for ALT and AST was downgraded on this basis in the GRADE assessment. Histological or elastographic outcomes were reported by only one trial [17], so effects on liver injury could not be assessed directly. Furthermore, the limited number of studies and inconsistent reporting of dosing and treatment duration precluded formal subgroup or meta-regression analyses, preventing evaluation of dose–response relationships for outcomes such as LDL and triglycerides. Future studies should aim to standardize reporting and investigate these factors to better define the therapeutic role of melatonin in MASLD.

### Conclusion

In this systematic review and meta-analysis of 11 controlled clinical trials (nine randomized and two non-randomized) including 548 participants, melatonin supplementation was associated with reductions in total and LDL cholesterol, IL-1, IL-6, TNF-α, body weight, and waist circumference, and with a lower proportion of participants with grade III steatosis at follow-up. No significant effect was observed on ALT, AST, GGT, ALP, triglycerides, HDL cholesterol, FBS, blood pressure, hs-CRP, or grade I–II steatosis in the primary analyses; reductions in liver enzymes and triglycerides appeared only in exploratory sensitivity analyses. Given the small number and size of the trials, the inclusion of non-randomized designs, high statistical heterogeneity, heterogeneous comparators and melatonin regimens, and incomplete safety reporting, these findings should be regarded as preliminary rather than as definitive evidence of efficacy. Melatonin may have a role as an adjunctive therapy in MASLD, but this requires confirmation in larger, adequately powered randomized trials with standardized comparators, dosing, outcome assessment, and adverse-event monitoring.

## Supplementary Material

Suppl 1 PubMed search strategy.

Suppl 2 Exploratory sensitivity analyses: trials omitted and reason for omission (values from the manuscript text and forest plots; reasons inferred from the forest plots).

Suppl 3 The summary of findings (SoF) constructed using GRADEPro GDT.

Suppl 4 (a) Risk of bias in the nine randomized trials assessed with the Cochrane RoB 2. (b) Risk of bias in the two non-randomized studies assessed with ROBINS-I.

Suppl 5 RoB 2 domain-level judgements and supporting information for the nine randomized trials.

Suppl 6 ROBINS-I domain-level judgements and supporting information for the two non-randomized studies.

Suppl 7 Forest plot showing post-sensitivity analysis of grade 1 hepatic steatosis.

Suppl 8 Forest plot showing post-sensitivity analysis of cholesterol levels.

Suppl 9 Forest plot showing post-sensitivity analysis of LDL levels.

Suppl 10 Forest plot showing the post-sensitivity analysis of ALT.

Suppl 11 Forest plot showing the post-sensitivity analysis of AST.

Suppl 12 Forest plot showing the post-sensitivity analysis of GGT.

Suppl 13 Forest plot showing the post-sensitivity analysis of triglycerides.

Suppl 14 Forest plot showing the post-sensitivity analysis of HDL.

Suppl 15 Forest plot showing post-sensitivity analysis of FBS levels.

Suppl 16 Forest plot showing post-sensitivity analysis of ALP levels.

Suppl 17 Adverse-event reporting in the included trials (extracted verbatim or paraphrased from each report).

## Data Availability

The authors declare that data analyzed in this study were obtained from publicly available sources.
