# Supplementary material for: Efficacy of Melatonin in Metabolic Dysfunction-Associated Steatotic Liver Disease: A Systematic Review and Meta-Analysis
Source: J Clin Med Res. 2026 Oct 23;18(10):687–703. doi: 10.14740/jocmr6688 (PMC13719654; doi:10.14740/jocmr6688)
Supplement: Supplementary file 1 [file jocmr-18-10-687-s001.docx]

**Suppl 1.** PubMed Search Strategy.

| ((((((((("Non-alcoholic Fatty Liver Disease"[Mesh]) OR (NAFLD)) OR (Nonalcoholic Fatty Liver Disease)) OR (Nonalcoholic Fatty Liver)) OR (Nonalcoholic Fatty Livers)) OR (Nonalcoholic Steatohepatitis)) OR (Nonalcoholic Steatohepatitides)) OR (((("Liver Cirrhosis"[Mesh]) OR (Hepatic fibrosis)) OR (Liver fibrosis)) OR (Hepatic Cirrhosis))) OR (((((((("Fatty Liver"[Mesh]) OR (Steatohepatitis)) OR (Steatohepatitides)) OR (Steatosis of Liver)) OR (Visceral Steatosis)) OR (Visceral Steatoses)) OR (Liver Steatosis)) OR (Liver Steatoses))) AND ("Melatonin"[Mesh]) | 246 |
| --- | --- |
