# Supplementary material for: Efficacy of Melatonin in Metabolic Dysfunction-Associated Steatotic Liver Disease: A Systematic Review and Meta-Analysis
Source: J Clin Med Res. 2026 Oct 23;18(10):687–703. doi: 10.14740/jocmr6688 (PMC13719654; doi:10.14740/jocmr6688)
Supplement: Supplementary file 2 [file jocmr-18-10-687-s002.docx]

**Suppl 2.** Exploratory sensitivity analyses: trials omitted and reason for omission (values from the manuscript text and forest plots; reasons inferred from the forest plots)

| **Outcome** | **Primary pooled estimate (all trials)** | **Trial(s) omitted** | **Reason for omission (criterion in Methods)** | **Estimate after omission** |
| --- | --- | --- | --- | --- |
| Grade I steatosis | RR 0.86 (0.39 to 1.92); I² = 71%; 3 trials | Bahrami 2020 | (i) CI did not overlap the pooled estimate | RR 0.64 (0.40 to 1.02), p = 0.06; I² = 15% |
| Total cholesterol | MD −16.80 mg/dL (−29.13 to −4.47); I² = 76%; 7 trials | Celinski 2014; Cichoz-Lach 2010; Gonciarz 2010 | (ii) essential phospholipids co-administered in both arms (Celinski, Cichoz-Lach) and (i) non-overlapping CIs; Gonciarz 2010: (i) estimate in the opposite direction | MD −12.07 mg/dL (−18.56 to −5.57), p = 0.0003; I² = 0% |
| LDL cholesterol | MD −52.4 mg/dL (−100.52 to −4.27); I² = 96%; 5 trials | Celinski 2014; Cichoz-Lach 2010 | (ii) essential phospholipids in both arms; (i) outlying estimates (−119 and −106 mg/dL) largely reflecting baseline imbalance | MD −10.45 mg/dL (−19.38 to −1.51), p = 0.02; I² = 0% |
| ALT | MD −7.97 IU/L (−17.43 to 1.49); I² = 84%; 10 trials | Cichoz-Lach 2010; Gonciarz 2012; Pakravan 2017 | Cichoz-Lach: (ii) essential phospholipids; Gonciarz 2012 and Pakravan: (i) CI not overlapping / opposite direction | MD −10.53 IU/L (−15.19 to −5.87); I² = 13% |
| AST | MD −5.23 IU/L (−14.12 to 3.67); I² = 91%; 10 trials | Gonciarz 2012; Chojnacki 2017; Mohammadi 2022 | Chojnacki: (ii) statin-treated population and outlying estimate; Gonciarz 2012 and Mohammadi: (i) opposite direction | MD −6.92 IU/L (−9.90 to −3.94); I² = 0% |
| GGT | MD −25.94 IU/L (−58.00 to 6.12); I² = 96%; 6 trials | Celinski 2014; Cichoz-Lach 2010; Gonciarz 2012 | (ii) essential phospholipids in both arms (Celinski, Cichoz-Lach); Gonciarz 2012: (i) opposite direction | MD −6.35 IU/L (−11.76 to −0.93); I² = 0% |
| Triglycerides | MD −22.03 mg/dL (−47.68 to 3.63); I² = 96%; 6 trials | Chojnacki 2017 | (ii) statin-treated population; (i) opposite direction | MD −34.58 mg/dL (−44.30 to −24.85); I² = 25% |
| HDL cholesterol | MD 1.71 mg/dL (−1.66 to 5.07); I² = 60%; 5 trials | Mohammadi 2022 | (i) CI did not overlap the pooled estimate | MD −0.40 mg/dL (−2.53 to 1.74); I² = 0% |
| ALP | MD −1.21 IU/L (−18.71 to 16.28); I² = 65%; 4 trials | Mojtahedi 2023 | (i) CI did not overlap the pooled estimate | MD 8.42 IU/L (−4.05 to 20.89); I² = 0% |
| Fasting blood sugar | MD −5.98 mg/dL (−13.30 to 1.34); I² = 52%; 3 trials | Mohammadi 2022 | (i) CI did not overlap the pooled estimate | MD −1.35 mg/dL (−9.09 to 6.39); I² = 0% |
