# Supplementary material for: Efficacy of Melatonin in Metabolic Dysfunction-Associated Steatotic Liver Disease: A Systematic Review and Meta-Analysis
Source: J Clin Med Res. 2026 Oct 23;18(10):687–703. doi: 10.14740/jocmr6688 (PMC13719654; doi:10.14740/jocmr6688)
Supplement: Supplementary file 3 [file jocmr-18-10-687-s003.docx]

**Suppl 3.** The summary of findings (SoF) constructed using GRADEPro GDT.

Author(s):

Question: Melatonin compared to Control for [NASH]

Setting:

Bibliography: [Melatonin] versus [Control] for [NASH]. Cochrane Database of Systematic Reviews [Year], Issue [Issue].

| **Certainty assessment** | | | | | | | **№ of patients** | | **Effect** | | **Certainty** | **Importance** |
| --- | --- | --- | --- | --- | --- | --- | --- | --- | --- | --- | --- | --- |
| **№ of studies** | **Study design** | **Risk of bias** | **Inconsistency** | **Indirectness** | **Imprecision** | **Other considerations** | **Melatonin** | **Control** | **Relative (95% CI)** | **Absolute (95% CI)** |  |  |
| **weight** | | | | | | | | | | | | |
| 2 | randomised trials | not serious | not serious | not serious | not serious | none | 73 | 69 | - | MD **6.25 lower** (15.43 lower to 2.93 higher) | ⨁⨁⨁⨁ High | IMPORTANT |
| **waist (cm)** | | | | | | | | | | | | |
| 2 | randomised trials | not serious | not serious | not serious | not serious | none | 73 | 69 | - | MD **3.06 lower** (6.72 lower to 0.6 higher) | ⨁⨁⨁⨁ High | IMPORTANT |
| **cholesterol** | | | | | | | | | | | | |
| 7 | randomised trials | not serious | serious^a^ | not serious | not serious | strong association | 187 | 166 | - | MD **16.8 lower** (29.13 lower to 4.47 lower) | ⨁⨁⨁⨁ High^a^ | IMPORTANT |
| **triglycerides** | | | | | | | | | | | | |
| 6 | randomised trials | not serious | serious^a^ | not serious | very serious^b^ | none | 157 | 154 | - | MD **22.03 lower** (47.68 lower to 3.63 higher) | ⨁◯◯◯ Very low^a,b^ | IMPORTANT |
| **ALT IU/L** | | | | | | | | | | | | |
| 10 | randomised trials | not serious | serious^a^ | not serious | not serious | publication bias strongly suspected^c^ | 284 | 248 | - | MD **7.97 lower** (17.43 lower to 1.49 higher) | ⨁⨁◯◯ Low^a,c^ | CRITICAL |
| **AST IU/L** | | | | | | | | | | | | |
| 10 | randomised trials | not serious | serious^a^ | not serious | not serious | publication bias strongly suspected^d^ | 284 | 248 | - | MD **5.23 lower** (14.12 lower to 3.67 higher) | ⨁⨁◯◯ Low^a,d^ | CRITICAL |
| **GGT IU/L** | | | | | | | | | | | | |
| 6 | randomised trials | not serious | serious^a^ | not serious | serious^b^ | none | 152 | 113 | - | MD **25.94 lower** (58 lower to 6.12 higher) | ⨁⨁◯◯ Low^a,b^ | IMPORTANT |
| **ALP IU/L** | | | | | | | | | | | | |
| 4 | randomised trials | not serious | not serious | not serious | not serious | none | 86 | 100 | - | MD **1.21 lower** (18.71 lower to 16.28 higher) | ⨁⨁⨁⨁ High | IMPORTANT |
| **Melatonin pg/ml** | | | | | | | | | | | | |
| 3 | randomised trials | not serious | not serious | not serious | not serious | none | 68 | 50 | - | MD **47.48 higher** (40.63 higher to 54.32 higher) | ⨁⨁⨁⨁ High | IMPORTANT |
| **bilirubin** | | | | | | | | | | | | |
| 2 | randomised trials | not serious | very serious^a^ | not serious | not serious | strong association | 38 | 38 | - | MD **0.15 higher** (0.05 higher to 0.25 higher) | ⨁⨁⨁◯ Moderate^a^ | IMPORTANT |
| **LDL mg/dl** | | | | | | | | | | | | |
| 5 | randomised trials | not serious | serious^a^ | not serious | not serious | strong association | 127 | 124 | - | MD **52.4 lower** (100.52 lower to 4.27 lower) | ⨁⨁⨁⨁ High^a^ | CRITICAL |
| **HDL mg/dl** | | | | | | | | | | | | |
| 5 | randomised trials | not serious | not serious | not serious | not serious | none | 127 | 124 | - | MD **1.71 higher** (1.66 lower to 5.07 higher) | ⨁⨁⨁⨁ High | CRITICAL |
| **IL-1 pg/ml** | | | | | | | | | | | | |
| 2 | randomised trials | not serious | not serious | not serious | not serious | strong association | 38 | 38 | - | MD **4.4 lower** (5.55 lower to 3.26 lower) | ⨁⨁⨁⨁ High | NOT IMPORTANT |
| **IL-6 pg/ml** | | | | | | | | | | | | |
| 2 | randomised trials | not serious | not serious | not serious | serious^b^ | strong association | 38 | 38 | - | MD **4.28 lower** (6.8 lower to 1.76 lower) | ⨁⨁⨁⨁ High^b^ | NOT IMPORTANT |
| **TNF-a pg/ml** | | | | | | | | | | | | |
| 3 | randomised trials | not serious | serious^a^ | not serious | not serious | strong association | 62 | 59 | - | MD **0.48 lower** (0.88 lower to 0.09 lower) | ⨁⨁⨁⨁ High^a^ | NOT IMPORTANT |
| **SBP mmHg** | | | | | | | | | | | | |
| 2 | randomised trials | not serious | not serious | not serious | not serious | none | 73 | 69 | - | MD **2.37 lower** (6.3 lower to 1.56 higher) | ⨁⨁⨁⨁ High | NOT IMPORTANT |
| **DBP mmHg** | | | | | | | | | | | | |
| 2 | randomised trials | not serious | serious^a^ | not serious | serious^b^ | none | 73 | 69 | - | MD **0.57 lower** (5.31 lower to 4.17 higher) | ⨁⨁◯◯ Low^a,b^ | NOT IMPORTANT |
| **hs-CRP ng/ml** | | | | | | | | | | | | |
| 2 | randomised trials | not serious | serious^a^ | not serious | serious^b^ | none | 73 | 69 | - | MD **1.01 lower** (2.25 lower to 0.23 higher) | ⨁⨁◯◯ Low^a,b^ | NOT IMPORTANT |
| **FBS mg/dl** | | | | | | | | | | | | |
| 3 | randomised trials | not serious | not serious | not serious | serious^b^ | none | 89 | 86 | - | MD **5.98 lower** (13.3 lower to 1.34 higher) | ⨁⨁⨁◯ Moderate^b^ | NOT IMPORTANT |
| **Grade I steatosis** | | | | | | | | | | | | |
| 3 | randomised trials | not serious | serious^a^ | not serious | not serious | none | 33/99 (33.3%) | 41/100 (41.0%) | **RR 0.86** (0.39 to 1.92) | **57 fewer per 1,000** (from 250 fewer to 377 more) | ⨁⨁⨁◯ Moderate^a^ | CRITICAL |
| **Grade II steatosis** | | | | | | | | | | | | |
| 3 | randomised trials | not serious | not serious | not serious | not serious | none | 18/99 (18.2%) | 24/97 (24.7%) | **RR 0.73** (0.44 to 1.21) | **67 fewer per 1,000** (from 139 fewer to 52 more) | ⨁⨁⨁⨁ High | CRITICAL |
| **Grade III steatosis** | | | | | | | | | | | | |
| 2 | randomised trials | not serious | not serious | not serious | serious^b^ | strong association | 3/64 (4.7%) | 13/62 (21.0%) | **RR 0.23** (0.07 to 0.77) | **161 fewer per 1,000** (from 195 fewer to 48 fewer) | ⨁⨁⨁⨁ High^b^ | CRITICAL |
| **GOS Normal** | | | | | | | | | | | | |
| 3 | randomised trials | not serious | serious^a^ | not serious | serious^b^ | none | 54/99 (54.5%) | 23/97 (23.7%) | **RR 2.82** (0.96 to 8.28) | **432 more per 1,000** (from 9 fewer to 1,000 more) | ⨁⨁◯◯ Low^a,b^ | NOT IMPORTANT |

**CI:** confidence interval; **MD:** mean difference; **RR:** risk ratio

#### Explanations

a. High heterogeneity and variable results across studies indicate some level of inconsistency across studies

b. Wide confidence intervals indicate some degree of imprecision

c. More studies on the positive side may indicate that studies with large positive effects are overrepresented as compared to studies with null or negative effects. Moreover, heterogeneity between the studies is also contributing.

d. More studies on the positive side may indicate that studies with large positive effects are overrepresented as compared to studies with null or negative effects. Moreover, heterogeneity between the studies is also contributing.
