# Supplementary material for: Efficacy of Melatonin in Metabolic Dysfunction-Associated Steatotic Liver Disease: A Systematic Review and Meta-Analysis
Source: J Clin Med Res. 2026 Oct 23;18(10):687–703. doi: 10.14740/jocmr6688 (PMC13719654; doi:10.14740/jocmr6688)
Supplement: Supplementary file 5 [file jocmr-18-10-687-s005.docx]

**Suppl 5. RoB 2 domain-level judgements and supporting information for the nine randomized trials.**

| **Trial (ref)** | **Domain** | **Judgement** | **Support for judgement** |
| --- | --- | --- | --- |
| Bahrami 2020 (3) | D1 Randomization process | Some concerns | Block randomization (blocks of six) with sealed envelopes; opacity/sequential numbering not stated. Baseline imbalance in diastolic BP (85.3 vs 78.7 mmHg, p = 0.012), marital status (p = 0.049) and, near-significantly, weight/BMI (p = 0.056/0.051). |
|  | D2 Deviations from intended interventions | Low risk | Double-blind; starch placebo of identical shape and colour; compliance checked by pill counts and phone calls. Analysis restricted to the 45 completers (no ITT) but deviations were minimal. |
|  | D3 Missing outcome data | Low risk | 5/50 (10%) did not complete (1 melatonin, 4 placebo); reasons ('put off', travel) unrelated to health status. |
|  | D4 Measurement of the outcome | Low risk | Laboratory outcomes; ultrasonographer blinded to allocation; automated sphygmomanometer. |
|  | D5 Selection of the reported result | Some concerns | Trial registered (IRCT2016061516123N8), but the registry outcome list was not checked; several tests (t-test/Mann–Whitney/ANCOVA) reported without a pre-specified analysis plan. |
|  | Overall | Some concerns | Some concerns (allocation concealment and baseline imbalance; analysis selection). |
| Celinski 2014 (4) | D1 Randomization process | High risk | 'Randomly assigned' but no sequence generation or concealment described; three-arm open design. Substantial baseline imbalance between the melatonin (group II) and Essentiale-only (group III) arms: LDL 197 ± 60 vs 256 ± 59 mg/dL, sex 15F/8M vs 11F/12M, age 36.2 vs 29.3 y, hypertriglyceridaemia 6 vs 11 patients. |
|  | D2 Deviations from intended interventions | Some concerns | No placebo and no blinding of participants or personnel; all arms received Essentiale forte. Per-protocol analysis; no information on adherence. |
|  | D3 Missing outcome data | Low risk | All 74 patients appear to have been analysed at 14 months; no attrition reported. |
|  | D4 Measurement of the outcome | Low risk | Objective laboratory outcomes (automated assays, ELISA, RIA); lack of blinding unlikely to affect them. |
|  | D5 Selection of the reported result | Some concerns | No protocol or registration; multiple biochemical parameters reported at two time points only; liver biopsy at follow-up performed in a self-selected subset (9/74). |
|  | Overall | High risk | High risk (randomization process). |
| Esmaeili 2021 (19) | D1 Randomization process | Low risk | Computer-generated sequence in blocks of four; allocation concealed from investigators until study completion; groups comparable at baseline (except total bilirubin). |
|  | D2 Deviations from intended interventions | Some concerns | Double-blind, matched placebo pearls. Crossover with only a 2-week washout after a 10 mg dose: a significant carry-over effect was detected for the primary (pruritus) outcome, which may also affect laboratory outcomes; analysis restricted to the 40 completers. |
|  | D3 Missing outcome data | Some concerns | 9/49 (18%) enrolled patients did not complete: 4 melatonin discontinuations for adverse reactions, 2 placebo discontinuations for GI upset, 3 for poor adherence — reasons plausibly related to the intervention. |
|  | D4 Measurement of the outcome | Low risk | Laboratory outcomes; assessors blinded. |
|  | D5 Selection of the reported result | Low risk | Registered (IRCT20180519039718N1); primary outcomes pre-specified; liver tests reported for every phase. |
|  | Overall | Some concerns | Some concerns (carry-over, attrition). Population = chronic liver disease with pruritus, not MASLD (indirectness). |
| Gonciarz 2010 (14) | D1 Randomization process | Some concerns | The 2010 report does not describe allocation; the 2012 extension report of the same 42 patients states random assignment in a 2:1 ratio, method and concealment not described. Baseline characteristics (age, sex, weight, enzymes) comparable. |
|  | D2 Deviations from intended interventions | Some concerns | Placebo given twice daily; blinding of participants/personnel not explicitly stated; identical lifestyle programme in both arms; completers analysed. |
|  | D3 Missing outcome data | Low risk | All 42 patients completed 12 weeks. |
|  | D4 Measurement of the outcome | Low risk | Objective laboratory outcomes (standard automated assays, RIA for melatonin). |
|  | D5 Selection of the reported result | Some concerns | No protocol or registration; medians with quartiles reported at four time points; ALT/AST/GGT/ALP/lipids all reported. |
|  | Overall | Some concerns | Some concerns. |
| Gonciarz 2012 (15) | D1 Randomization process | Some concerns | Same randomized cohort as Gonciarz 2010 (2:1 random allocation stated; method/concealment not described). |
|  | D2 Deviations from intended interventions | Some concerns | Placebo-controlled; blinding not explicitly described; lifestyle programme in both arms; completers analysed. |
|  | D3 Missing outcome data | Some concerns | 3/15 (20%) control patients dropped out (reasons not given) versus 0/30 in the melatonin arm — differential attrition of unknown cause. |
|  | D4 Measurement of the outcome | Low risk | Objective laboratory outcomes. |
|  | D5 Selection of the reported result | Some concerns | No protocol; results at weeks 18, 24 and after a 12-week off-treatment follow-up; BMI data 'not included' because not significant. |
|  | Overall | Some concerns | Some concerns. |
| Pakravan 2017 (11) | D1 Randomization process | Some concerns | Computer software ('Random Allocation'); concealment not described; baseline characteristics comparable. |
|  | D2 Deviations from intended interventions | Low risk | Double-blind; placebo manufactured identically to melatonin tablets; same diet/exercise advice in both arms; 3/100 lost. |
|  | D3 Missing outcome data | Some concerns | 3/100 lost to follow-up (1 vs 2, 'unwilling to continue') for the biochemical outcomes; however the steatosis-grade table is based on 40 + 41 patients without explanation of the 16 missing cases. |
|  | D4 Measurement of the outcome | Low risk | Blinded expert radiologist for ultrasound grading; laboratory outcomes. |
|  | D5 Selection of the reported result | High risk | No registration/protocol. Internal inconsistencies in the report (abstract: melatonin thrice daily for 3 months; methods: twice daily for 6 weeks; dose in mg never stated; table footnotes refer to 'oral cumin capsule'); adverse-event rates 'data not shown'; denominators of the steatosis outcome differ from the analysed population. |
|  | Overall | High risk | High risk (selection of the reported result / reporting inconsistencies). |
| Chojnacki 2017 (18) | D1 Randomization process | Some concerns | 'Randomly allocated' with no description of sequence generation or concealment; baseline enzymes and lipids comparable. |
|  | D2 Deviations from intended interventions | Some concerns | Placebo from the same manufacturer given at the same times; blinding of participants/personnel not explicitly stated; all patients on the same statin dose and 1600-kcal diet. |
|  | D3 Missing outcome data | Some concerns | Attrition not reported; percentages in the results imply denominators of 32 (group I) and 27 (group II) rather than 30 and 30. |
|  | D4 Measurement of the outcome | Low risk | Objective laboratory outcomes at 2, 4 and 6 months. |
|  | D5 Selection of the reported result | Some concerns | No protocol or registration; outcomes (AST, ALT, GGT, ALP, lipids) reported as pre-specified but only as means with p-values at selected time points. |
|  | Overall | Some concerns | Some concerns. Population = statin-treated hyperlipidaemia with elevated aminotransferases (BMI > 30 excluded), MASLD not an inclusion criterion (indirectness). |
| Mohammadi 2022 (12) | D1 Randomization process | Some concerns | Block randomization list (sealedenvelope.com) with sealed envelopes; baseline weight differed significantly between arms (melatonin 74.9 vs control 89.4 kg, p < 0.001), addressed by ANCOVA. |
|  | D2 Deviations from intended interventions | Low risk | Double-blind with two/three placebos per arm; patients and physicians unaware of allocation; telephone monitoring of adherence; 140/142 analysed. |
|  | D3 Missing outcome data | Low risk | 2/142 (1.4%) withdrawals, both in the metformin arm. |
|  | D4 Measurement of the outcome | Low risk | Blinded sonographer; laboratory outcomes. |
|  | D5 Selection of the reported result | Low risk | Registered (IRCT2016040318124N3); primary (AST, ALT) and secondary outcomes stated a priori and all reported. |
|  | Overall | Some concerns | Some concerns (baseline imbalance in weight). |
| Mojtahedi 2023 (17) | D1 Randomization process | Low risk | Block randomization (1:1:1:1) stratified by age, sex and steatosis grade; allocation sequence generated by a research assistant not involved in the study and concealed from investigators and participants until the final analysis; four arms balanced at baseline (all p > 0.10). |
|  | D2 Deviations from intended interventions | Low risk | Double-blind: drugs and placebo pre-packaged and encoded by trained staff in indistinguishable packages; adherence monitored by telephone, monthly interviews and pill counts; 120/120 randomized patients analysed. A 500-kcal-deficit weight-loss diet was prescribed in all arms. |
|  | D3 Missing outcome data | Low risk | No drop-outs (CONSORT diagram: 30/30 analysed in every arm). |
|  | D4 Measurement of the outcome | Low risk | Steatosis and fibrosis by FibroScan/CAP read by a hepatologist masked to allocation; laboratory outcomes; anthropometry by a trained kinanthropometrist. |
|  | D5 Selection of the reported result | Low risk | Registered (IRCT201407201155N21) and reported per CONSORT; primary (steatosis, fibrosis, ALT, AST, ALP) and secondary outcomes stated a priori and all reported with pre- and post-treatment values; ANCOVA adjusted for age, sex and baseline BMI as planned. |
|  | Overall | Low risk | Low risk. |
