# Supplementary material for: Efficacy of Melatonin in Metabolic Dysfunction-Associated Steatotic Liver Disease: A Systematic Review and Meta-Analysis
Source: J Clin Med Res. 2026 Oct 23;18(10):687–703. doi: 10.14740/jocmr6688 (PMC13719654; doi:10.14740/jocmr6688)
Supplement: Supplementary file 6 [file jocmr-18-10-687-s006.docx]

**Suppl 6. ROBINS-I domain-level judgements and supporting information for the two non-randomized studies.**

| **Trial (ref)** | **Domain** | **Judgement** | **Support for judgement** |
| --- | --- | --- | --- |
| Cichoz-Lach 2010 (13) | D1 Confounding | Serious | Patients 'divided into three groups' without randomization; no baseline demographic data per group (only overall age ranges and sex); no adjustment for confounders; the melatonin arm had higher baseline ALT and lower AST than the placebo arm. |
|  | D2 Selection of participants | Moderate | Selection process into the trial and into groups not described. |
|  | D3 Classification of interventions | Low | Interventions clearly defined (melatonin 2 × 5 mg vs placebo, both with Essentiale forte). |
|  | D4 Deviations from intended interventions | Moderate | No blinding described; co-intervention (Essentiale forte with vitamins) identical in all arms; adherence not reported. |
|  | D5 Missing data | Low | All 45 patients apparently measured at 4 weeks; no missing data reported. |
|  | D6 Measurement of outcomes | Low | Objective laboratory outcomes; assessor blinding not reported. |
|  | D7 Selection of the reported result | Moderate | No protocol; results expressed as mean ± standard error; 4-week data only. |
|  | Overall | Serious | Serious risk (confounding). |
| Gonciarz 2013 (16) | D1 Confounding | Critical | Uncontrolled before–after study: 16 overweight patients with biopsy-proven NASH received melatonin 2 × 5 mg/day for 28 days; the only comparison group was 15 lean, untreated healthy subjects (BMI 23.1 vs 28.6 kg/m²; 7/8 vs 11/5 M/F; 'the study was not balanced with respect to distribution of gender and weight'). There is no untreated MASLD group, so any between-group contrast reflects disease status, not melatonin, and within-patient changes are unadjusted for regression to the mean or co-interventions. |
|  | D2 Selection of participants | Serious | Patients recruited from one gastroenterology department; selection of the 16 patients and of the healthy controls not described (Hp-positive patients and those with gastric mucosal injury excluded). |
|  | D3 Classification of interventions | Low | Intervention clearly defined (melatonin 5 mg at 09:00 and 21:00 for 28 days). |
|  | D4 Deviations from intended interventions | No information | No information on diet, exercise or other co-interventions during treatment; no blinding (open treatment). |
|  | D5 Missing data | Low | All 16 patients appear to have been measured at day 28; no missing data reported. |
|  | D6 Measurement of outcomes | Low | Objective laboratory outcomes (automated assays, RIA); enzymes reported as medians with quartiles. |
|  | D7 Selection of the reported result | Moderate | No protocol or registration; enzymes are a secondary covariate; results reported as medians and percentage changes. |
|  | Overall | Critical | Critical risk (confounding: no comparable untreated MASLD control group). This study does not satisfy eligibility criterion (1) 'concurrent control group in adults with MASLD' and contributes to no pooled estimate (the pooled melatonin-level analysis comprises Cichoz-Lach, Gonciarz 2010 and Celinski: n = 68/50) — consider excluding. |
