# Supplementary material for: Efficacy of Melatonin in Metabolic Dysfunction-Associated Steatotic Liver Disease: A Systematic Review and Meta-Analysis
Source: J Clin Med Res. 2026 Oct 23;18(10):687–703. doi: 10.14740/jocmr6688 (PMC13719654; doi:10.14740/jocmr6688)
Supplement: Supplementary file 17 [file jocmr-18-10-687-s017.docx]

**Suppl 17. Adverse-event reporting in the included trials (extracted verbatim or paraphrased from each report).**

| **Trial (ref)** | **Adverse events assessed/reported?** | **Findings (melatonin vs control)** | **Withdrawals attributed to adverse effects** | **Serious adverse events** |
| --- | --- | --- | --- | --- |
| Bahrami 2020 (3) | Yes | 'No adverse effect was observed' after 12 weeks of 6 mg/day (0/24 vs 0/21). | None (5 dropouts for reasons unrelated to adverse effects) | None reported |
| Celinski 2014 (4) | Yes | 'No side effects of melatonin and tryptophan were observed'; no excessive sleepiness or dizziness (0/23 melatonin, 0/23 Essentiale-only). | None | None reported |
| Esmaeili 2021 (19) | Yes (Naranjo scale) | Melatonin phase: 4 adverse drug reactions (drowsiness 2, headache 1, gastrointestinal disturbance 1), all 'probable'; placebo phase: 5 reports of GI upset. Drowsiness was the most common effect. | 4 (melatonin) and 2 (placebo) of 49 enrolled | None reported |
| Cichoz-Lach 2010 (13) | No | Not reported. | Not reported | Not reported |
| Gonciarz 2010 (14) | Yes | 'No significant side effects'; slight somnolence in 12/30 melatonin-treated patients during the first 4 weeks; no insomnia; control arm not reported. | None | None reported |
| Gonciarz 2012 (15) | Yes | 'No patient complained somnolence and no significant side-effects were observed' during weeks 12–24; tolerability described as excellent. | None (3 control dropouts, reasons not given) | None reported |
| Pakravan 2017 (11) | Partly | 'No serious side effects were observed, and the rates of other side effects were similar between the two groups (data not shown)'. | None (3 patients 'unwilling to continue') | None |
| Chojnacki 2017 (18) | Yes | 'All drugs were well tolerated'; mild morning fatigue in 6/30 melatonin-treated patients during the first 2 weeks; 0/30 placebo. | None reported | None reported |
| Mohammadi 2022 (12) | Monitoring described, results not reported | Telephone monitoring for drug intolerance, drowsiness, excess sleep and GI upset described in Methods; no adverse-event results reported (2 withdrawals, both in the metformin arm). | None in the melatonin arm | Not reported |
| Mojtahedi 2023 (17) | Partly (serious adverse effects only) | 'No serious adverse effects were observed or reported among participants throughout the trial'; non-serious adverse events not reported by arm. | None (no drop-outs in any arm) | None |
| Gonciarz 2013 (16) | Yes | 'During the treatment period, no patient complained of somnolence and no side effects of melatonin were observed' (16 patients, 28 days). | None | None reported |

GI: gastrointestinal. Nine trials reported on adverse events (Pakravan 2017 and Mojtahedi 2023 only that no serious adverse effects occurred), Mohammadi 2022 described monitoring without reporting results, and Cichoz-Lach 2010 reported nothing. No trial reported adverse events systematically by arm with denominators, so pooling was not attempted.
