# Supplementary material for: Efficacy of Melatonin in Metabolic Dysfunction-Associated Steatotic Liver Disease: A Systematic Review and Meta-Analysis
Source: J Clin Med Res. 2026 Oct 23;18(10):687–703. doi: 10.14740/jocmr6688 (PMC13719654; doi:10.14740/jocmr6688)
Supplement: Supplementary file 24 [file jocmr-18-10-687-s013.docx]

**Suppl 13.** Forest plot showing the post-sensitivity analysis of triglycerides
